# Mobile-Based Nutrition and Child Health Monitoring to Inform Program Development: An Experience From Liberia

**DOI:** 10.9745/GHSP-D-16-00189

**Published:** 2016-12-23

**Authors:** Agnes Guyon, Ariella Bock, Laura Buback, Barbara Knittel

**Affiliations:** a John Snow, Inc., Arlington, VA, USA.; b UNICEF West and Central Africa Regional Office, Dakar, Senegal.

## Abstract

**Background:**

Implementing complex nutrition and other public health projects and tracking nutrition interventions, such as women's diet and supplementation and infant and young child feeding practices, requires reliable routine data to identify potential program gaps and to monitor trends in behaviors in real time. However, current monitoring and evaluation practices generally do not create an environment for this real-time tracking. This article describes the development and application of a mobile-based nutrition and health monitoring system, which collected monitoring data on project activities, women's nutrition, and infant and young child feeding practices in real time.

**Program Description:**

The Liberia Agricultural Upgrading Nutrition and Child Health (LAUNCH) project implemented a nutrition and health monitoring system between April 2012 and June 2014. The LAUNCH project analyzed project monitoring and outcome data from the system and shared selected behavioral and programmatic indicators with program managers through a short report, which later evolved into a visual data dashboard, during program-update meetings. The project designed protocols to ensure representativeness of program participants.

**Findings:**

LAUNCH made programmatic adjustments in response to findings from the monitoring system; these changes were then reflected in subsequent quarterly trends, indicating that the availability of timely data allowed for the project to react quickly to issues and adapt the program appropriately. Such issues included lack of participation in community groups and insufficient numbers of food distribution points. Likewise, the system captured trends in key outcome indicators such as breastfeeding and complementary feeding practices, linking them to project activities and external factors including seasonal changes and national health campaigns.

**Conclusion:**

Digital data collection platforms can play a vital role in improving routine programmatic functions. Fixed gathering locations such as food distribution points represent an opportunity to easily access program participants and enable managers to identify strengths and weaknesses in project implementation. For programs that track individuals over time, a mobile tool combined with a strong database can greatly improve efficiency and data visibility and reduce resource leakages.

## Abstract (precis)

Monitoring behavior using mobile phones at food distribution points allowed managers to rapidly adapt project activities. Self-reported breastfeeding, complementary feeding, and use of insecticide-treated nets improved. Applying the same methodology at the household level proved unsuccessful.

## BACKGROUND

Proper nutrition during the 1,000 days between the time of conception and a child's second birthday sets the foundation for all the days that follow; it is considered one of the best interventions for ensuring optimum physical and cognitive development. The *Preventing Malnutrition in Children Under 2 Approach* (PM2A), used by the United States Agency for International Development (USAID) Office of Food for Peace, provides a blanket food supplementation approach to pregnant and lactating women and children under 2 years of age regardless of nutritional status.^1^^–^^3^

Implementing complex projects and tracking nutrition interventions, such as women's diet and supplementation nutrition and infant and young child feeding practices, requires reliable routine data to identify potential program gaps and monitor trends in behaviors in real time. However, current monitoring and evaluation practices used in nutrition-related projects generally do not create an environment for this real-time tracking.^4^ The traditional baseline, midterm, and endline household-based surveys, used by most PM2A programs to track behavioral practices of program participants, are unable to provide routine and detailed nutritional information to effectively monitor project performance or trends in practices between time periods. In addition to the high costs and resources needed to reach individual households, these exhaustive surveys typically provide only general data that limit a manager's ability to identify problems at the implementation level or allow for rapid programmatic adjustments. Conversely, routine reporting often established for monitoring activities typically collects process data, such as people reached or trained and numbers of participants receiving foods; it fails to capture information on how the program is used by the intended population or whether targeted behaviors are changing over time.^5^ Likewise, supportive supervision visits, another monitoring practice used by projects, often focus on micro-level behavior changes (i.e., the particular community or providers) and do not capture information in a systematic way, thereby restricting a program's ability to generalize.

The purpose of this article is to describe the development and application of a mobile-based nutrition and health monitoring system, developed under the Liberia Agricultural Upgrading Nutrition and Child Health (LAUNCH) project, funded by the USAID Office of Food for Peace. The system collected monitoring data on project activities, women's nutrition, and infant and young child feeding practices in real time. This article demonstrates how fixed programmatic points of contact, such as food distribution points (FDPs), can be used as an affordable and pragmatic alternative to household-based surveys to provide similarly useful and reliable data on key nutritional and health indicators necessary for many programmatic decisions.

## LAUNCH PROJECT

The LAUNCH project, implemented by ACDI/VOCA and its partners Project Concern International, John Snow, Inc. (JSI), and Making Cents International, worked in 6 selected districts in Bong and Nimba counties in Liberia from June 2011 to June 2016. Selected districts included Gbor (Nimba), Salala (Bong), Sanoyea (Bong) Wee Gbey Mahn (Nimba), Yarpea Mahn (Nimba), and Zoe Gbao (Nimba). The project worked closely with the Ministry of Agriculture and the Ministry of Health and Social Welfare, and their respective county offices. The project applied an integrated PM2A approach to improve food security and reduce chronic malnutrition of vulnerable women and children under age 5. This entailed providing a comprehensive package of interventions that included supplementary feeding for pregnant or lactating women and children under 2 years (i.e., 6–23 months); increasing availability of and access to food through sustainable livelihoods interventions that included use of farmer groups, which cultivated group block farms using improved technology; strengthening facility and community health and nutrition services, including implementation of the Essential Nutrition Actions framework; and increasing access to primary education.^6^^–^^8^

As part of the program, LAUNCH established FDPs where pregnant or lactating women and mothers of children ages 6–23 months from the selected districts collected food rations such as corn soya blend, bulgur, vegetable oil, and yellow split peas on a monthly basis. These same women were also members of LAUNCH community Care Groups that met twice a month to discuss and receive trainings on proper health and nutrition practices. (These women then distilled the information and practices in smaller community “mother groups.”) In addition, the project worked closely with health care providers in government facilities, government-recognized community volunteers, general community heath volunteers, and traditional trained midwives in the same communities to promote key health and nutritional behavior messages.

The LAUNCH project established food distribution points where women and children collected food rations and held community Care Groups that met bi-monthly to discuss health and nutrition practices.

### Nutrition and Health Monitoring System

The LAUNCH project operated a nutrition and health monitoring system between April 2012 and June 2014 (9 quarters) to provide routine and detailed information to monitor performance and track key programmatic outcome indicators in the project's communities (Box 1). The project collected information on a rolling basis through interviews with a random sample of program participants after they received their monthly food rations. Project food monitors collected data using mobile devices and uploaded it to a cloud-based server, providing program managers with real-time program management metrics. The project analyzed the data each quarter and shared selected behavior and programmatic indicators with program managers through a short report, which later evolved into a visual data dashboard, during program-update meetings.

BOX 1. **Key Indicators Captured by the LAUNCH Project's Nutrition and Health Monitoring System**

**Required indicators by the U.S. Agency for International Development Office of Food for Peace:**

- Percentage of infants ages 0–5 months who are exclusively breastfed
- Percentage of children ages 6–23 months who receive minimum acceptable diet
- Percentage of caregivers demonstrating proper food hygiene behaviors
- Percentage of households reporting an outbreak of diarrhea

**Program monitoring indicators:**

- Mother's nutrition and eating practices (e.g., variety, frequency, corn soya blend consumption, and iron-folic acid supplementation)
- Young child feeding practices (e.g., corn soya blend consumption, variety and frequency of complementary food, who feeds the child and how)
- Insecticide-treated net use (i.e., malaria and anemia prevention)
- Handwashing and hygienic food preparation
- Corn soya blend storage and preparation
- Participation in LAUNCH community groups
- Duration of rations
- Satisfaction with rations

LAUNCH operated a nutrition and health monitoring system using mobile devices and a cloud-based server to provide real-time program management metrics.

Key behavioral indicators captured by the monitoring system included variety and frequency of women's nutrition practices; iron-folic acid supplementation during pregnancy; percentage of infants (ages 0–5 months) who are exclusively breastfed; variety and frequency of complementary feeding practices for young children (ages 6–23 months); percentage of young children (ages 6–23 months) who receive a minimum acceptable diet; percentage of caregivers demonstrating proper food hygiene behaviors; and percentage of households reporting an outbreak of diarrhea. Programmatic indicators included distance traveled to FDPs, corn soya blend storage and preparation, and participation in LAUNCH community groups. Duration of and satisfaction with rations were added as program indicators in October 2013.

From April 2012 to September 2013 (6 quarters), the monitoring system relied on interviews conducted by food monitors with program participants at FDPs, before programmatic requirements shifted the point of contact to the household level (but still conducted by the same food monitors). A modified system continued to function for another 3 quarters (October 2013 to June 2014) until the Ebola outbreak led to the cancellation of food distribution and a substantial change in programming, effectively ending project monitoring related to food distribution and nutrition.

The monitoring system relied on interviews conducted with program participants at food distribution points, and then shifted to using household surveys.

### Questionnaire and Mobile Data Platform

LAUNCH developed a questionnaire that included both programmatic questions (Box 2) and nutritional behavior questions based on 24-hour recall (Box 2). The questions were based on the Demographic and Health Surveys and followed recommended indicators from the World Health Organization (WHO) (e.g., the minimum acceptable diet composite indicator). Survey administrators asked the questions using prescriptive WHO guidelines.^9^^–^^10^ The questionnaire was limited to 25 questions to keep interview times to less than 10 minutes, to avoid overburdening program participants and food monitors who had other responsibilities at the FDPs. When the point of contact shifted to the household level, the project added 15 more questions; this increase did not have any significant impact on implementation.

BOX 2. **Sample Questions Included in the Nutrition and Health Mobile Monitoring System**

**Sample Programmatic Questions:**

Q22: Are you part of a LAUNCH care group or mother group? Possible responses:

□ Care group (lead mother)

□ Mother group

□ None → Skip to #26

Q39: How do you store the CSB? Possible responses:

□ In a plastic bag

□ In a cloth

□ In a bucket with a cover

□ In a jar with a cover

□ NA—First time receiving

**Sample Nutritional Behavior Questions:**

Q13: Yesterday, did you feed your child Tete Water (breast milk)? Possible responses:

□ Yes

□ No

Q15: Yesterday, did you feed your child any milk products, such as powdered milk, animal milk, or yogurt? Possible responses:

□ Yes

□ No → Skip to #17

Q17: Yesterday, how many times did you feed your child? Possible responses:

□ One time

□ Two times

□ Three times

□ Four times

□ More than four times

Abbreviations: CSB, corn soya blend; NA, not applicable.

**FIGURE 1:**
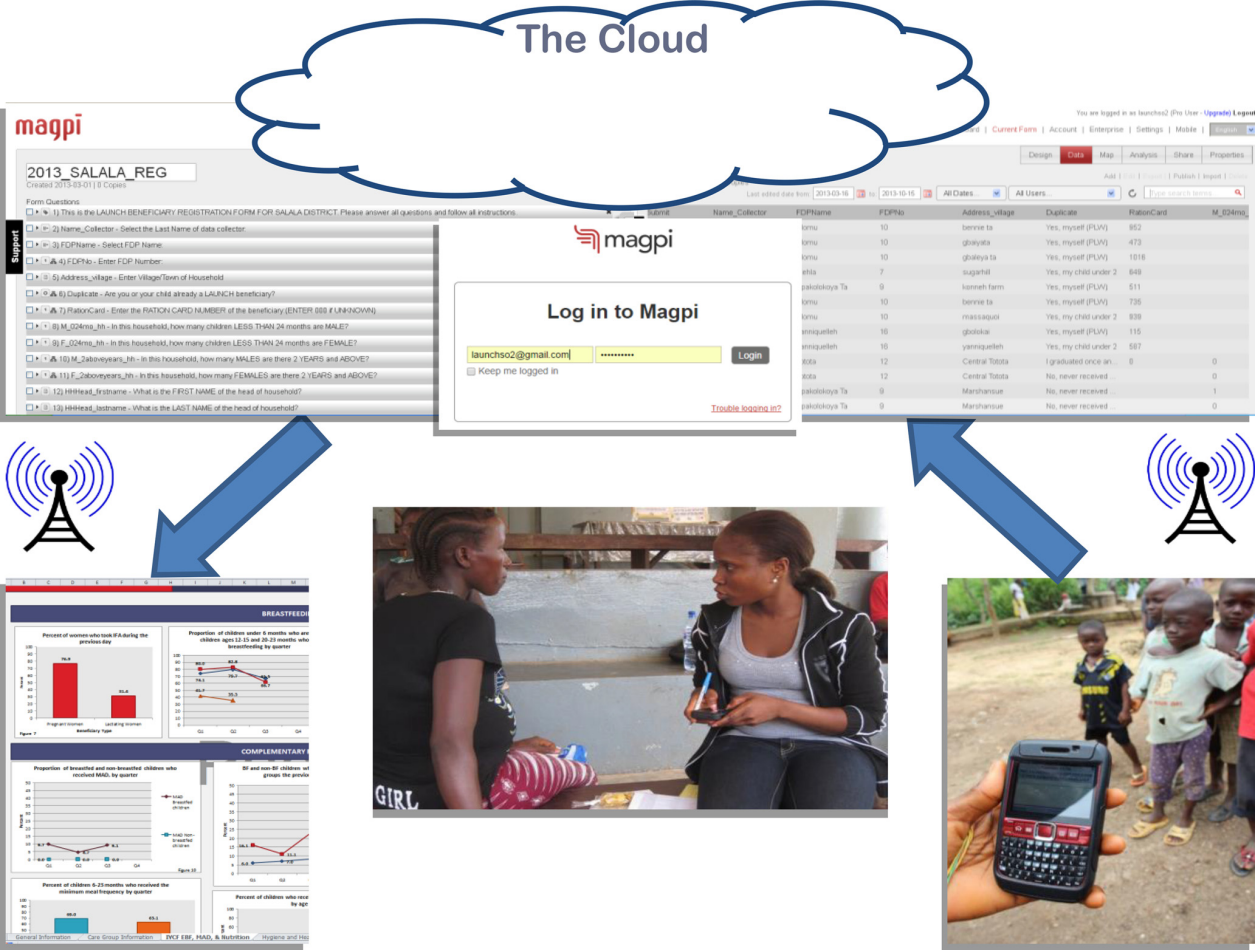
Diagram of LAUNCH Project's Nutrition and Health Monitoring System in Liberia Field staff used mobile smartphones (right) to collect monitoring data, from either food distribution points or households, which were then transferred to a cloud-based server for storage when an Internet connection was available (top center). Project managers then downloaded, analyzed, and presented the data in visual format (left) to inform decision making at meetings.

The project chose to use smartphones (Nokia phones with a J2ME platform, later replaced with Samsung Android-based phones as the project expanded) and Magpi (formerly called EpiSurveyor), a digital data collection platform, to collect data. Using this platform, data from both the FDPs and household interviews were automatically transferred to a cloud-based server for storage and immediately available to download for analysis. Taking advantage of the increased power and availability of smartphone technology, digital data collection enables a user to collect data via mobile smartphone or tablet computer and store data offline (i.e., out of mobile network or Wi-Fi range). Once the user is back online, the data can be uploaded and saved to the cloud-based server. Digital data collection is generally associated with a cloud-based platform where a user designs data collection forms using a web-based application, and then downloads them to compatible devices. When the forms are filled in, the data are uploaded to a server using mobile phone data networks or Wi-Fi. The data can then be easily accessed online and are immediately available for supervisors to monitor and identify errors, then subsequently use for analysis and reporting (Figure 1).

Digital data collection applications use the advantages of computer programming to ensure better data quality and accuracy during data collection by making it possible to require respondents to answer a question before proceeding to the next question, skipping a question based on a response in a previous question, or adding constraints such as minimum and maximum values for numeric responses. Additionally, because data are entered directly into the mobile smartphone, the need for a separate data entry step is removed, reducing potential for error due to handwriting issues or mistakes during the data entry process. Data access privileges can also be customized in digital data collection, giving access for editing or downloading data to only those named, which reduces the opportunity to tamper with data and helps to ensure data integrity.

While many different digital data collection tools and applications existed, the LAUNCH project selected Magpi because it was user-friendly, easy to learn, required little to no programming experience to use, and was relatively inexpensive. Furthermore, Magpi had successfully been used in other JSI projects in Liberia and this experience proved that it could effectively avoid many of the challenges associated with paper-based data collection and that it was feasible for use in low-resource settings.

The LAUNCH food monitors were responsible for the task of data collection. They participated in a week-long training to introduce them to the mobile devices, test the tool, and practice using Magpi. This was followed by frequent supervision visits, particularly during the first 6 months of implementation, as the food monitors grew accustomed to their additional responsibility. At the central level, data were reviewed online, and then downloaded into Microsoft Excel for further analysis. In addition to participants beings selected at random, no personal information was collected. Once in Excel, data were then stored on the program computers and server, with the benefit of the original data backed up online. Processes, roles, and responsibilities were adapted throughout implementation, including restricting online access to the raw data to a select few. Box 3 includes a list of important factors to consider during the start-up phase of a digital data collection activity, based on the experiences of the LAUNCH project.

BOX 3. **Factors to Consider at the Beginning of a Digital Data Collection Activity**

Throughout its implementation, the LAUNCH project faced various challenges with its mobile phone-based monitoring system related to equipment and asset management, Internet connectivity, and staff capacity. Based on LAUNCH's experience, the following factors are important to consider during the start-up phase of a digital data collection activity:

- **Equipment and fees:** The initial start-up needs and cost for the LAUNCH system included a 1-year subscription to Magpi, 8 smartphones, and small additional costs for phone accessories including car chargers, airtime, and backpacks to protect the phones. Once the system was shown to function and bring results, additional smartphones were purchased.
- **Asset management:** Protecting the phones from weather damage, loss, or theft required forethought and problem solving. LAUNCH instituted strict policies for giving the phones to staff, and provided plastic bags and backpacks to staff so that they could protect the devices during field conditions and inclement weather.
- **Internet connectivity and/or network access:** While no Internet connection is required to collect data, an early challenge involved locating hot spots or network areas with a strong network signal (i.e., 2 bars or more) so data could be transmitted. Internet connection was also required when program managers or supervisors were creating and managing forms or accessing data on the Magpi website. The program (both the mobile application and website) is designed to be used in lower-resource settings; however, Internet speeds vary from country to country. In general, it took about 240 kb to load the main Magpi home page.
- **Training and mentoring staff in digital data collection:** Baseline levels of phone and computer literacy of staff varied. Training staff on devices (e.g., computers and smartphone devices) and how to conduct digital data collection was critical and helped to ensure consistency across enumerators. Staff time was initially dedicated for training data collectors and providing close supervision during the rollout phase. After the initial training and start-up, staff time was required for routine data collection activities, supervision, troubleshooting, and analysis.

LAUNCH food monitors were responsible for collecting data using the Magpi data collection tool.

### Participant Selection, Analysis, and Confidentiality

The nutrition and health monitoring system aimed to provide meaningful programmatic-level results that were statistically representative for each target group, as one would obtain through a household survey. Similar steps were therefore applied when designing the methodology. Using the sampling formula for cross-sectional surveys and the targeted population numbers used in the PM2A calculations, LAUNCH calculated the desired number of interviews needed for each quarter. The confidence level and margin of error for FDP-based results for all groups combined were set at 95% and 5%, respectively, whereas 90% and 8%, respectively, were used for the subgroups (pregnant women, lactating women with children under 6 months, and mothers of children ages 6–23 months). Based on these calculations, the project required a minimum of 377 interviews per quarter—including 102 pregnant women, 102 lactating women with children under 6 months, and 105 mothers of children ages 6–23 months. A data collection plan took into account specific logistical considerations, including frequency of point of contact (i.e., FDPs), existing and available staff (i.e., food monitors), staff responsibilities, and availability of project vehicles and motorbikes for movement between sites. For logistical purposes, the quarterly goal for food monitors was set at 400 interviews total.

Interviews were limited to program participants, as many of the questions were related to their behavior and based on a 24-hour recall. During the first 18 months of implementation, this meant conducting interviews with those who were physically present at FDPs to receive their food rations, excluding those who sent an alternate or those who did not attend the distribution in a given month. This was recognized as a potential limitation of the system; those sending alternates could potentially have very different feeding habits or other challenges that would not be captured and would therefore produce results that were not representative of the desired population (i.e., program participants). Discussions with community-level project staff indicated that alternates represented a small portion (less than 10%) of those collecting the food rations. This was later confirmed during a project assessment on commodity distribution and defaulters.

The project designed protocols to ensure representativeness of program participants. At each FDP, field staff randomly interviewed 5 women over the course of the day—2 mothers of children ages 6–23 months, 1 pregnant woman, 1 lactating woman with a child under 6 months, and then either another pregnant woman or lactating woman with a child under 6 months for the fifth interviewee. The composition of the sample was developed to align with the overall distribution of program participants obtained from the project's commodity logistics management information system (CLMIS)—approximately 75% mothers of children ages 6–23 months and 25% pregnant or lactating women (half were assumed to be pregnant and the other half lactating). Interviews occurred throughout the 4- to 6-hour period that the FDP operated with field staff approaching program participants for interviews using the “spinning the pen” technique to help ensure randomization. To reduce response bias and avoid any notion that food rations would depend of their answers, interviews were conducted after program participants received their monthly food ration. During the analysis phase, probability weights were applied to the interview records so that results would be representative of all program participants who received food rations in the quarter. Program data on the number of participants receiving food from the CLMIS facilitated these weights.

To reduce response bias and avoid any notion that food rations would depend on their answers, interviews were conducted after program participants received their monthly food ration.

For programmatic reasons, the point of contact shifted from the FDPs to the household level in October 2013, and slight adjustments were made to the sampling framework and implementation. To reduce some of the extra costs and resources required for the additional travel, the margin of error for results was increased to 6%, thereby reducing the overall sample size to 200. Additionally, the pre-conditions of attendance at the FDPs or receipt of food rations during the previous month were removed. Instead, each month, field staff visited a list of households located in 8 to 12 communities that were randomly generated from the project's registered food recipient database. To provide a geographically representative picture of program activities, communities were linked to different FDPs that rotated each month, covering approximately 25% of the FDPs each quarter. The list generated from CLMIS consisted of an equal mix of pregnant or lactating women and mothers with children ages 6–23 months, plus a backup list of names, to ensure that meaningful programmatic-level results for each target group could still be calculated. Analysis of nutrition indicators followed WHO recommendations—for the minimum acceptable diet, breastfeeding practices, frequency of feeding per age group and food diversity—and were analyzed and computed to obtain the minimum acceptable diet composite indicator. Probability weights were also applied during the analysis so that results would be representative of all program participants. Project staff analyzed findings using SPSS v16 in 2012–2013 and STATA 12 in 2014.

Several steps were also taken to protect confidentiality. Each interviewee provided verbal consent before answering questions. No personal identifying information, such as name or age, was collected; the questionnaire collected only program ration card numbers—a unique identifier provided to each household generated by the CLMIS, so that secondary analysis could be carried out. Furthermore, as part of the basic application and web-based server, Magpi provided advance security measures so the data remained secure in both the mobile application and cloud-based server. Food monitors could only view data gathered on their specific phone, while only a select few in Monrovia had the password to access the full data online.

### Data Use Approach

The nutrition and health monitoring system provided LAUNCH with timely information that enabled program managers to track program performance in real time. Each quarter, project staff downloaded, cleaned, and analyzed data collected at the FDPs from Magpi. Staff then presented key program indicators in a short report for program managers to review. The report included a description of the interviewed respondents (i.e., background characteristics and distance to FDP sites), as well as program data on women's nutrition, breastfeeding and complementary feeding practices, hygiene, and care group participation. The report presented indicators across time periods, allowing for comparisons and trends. It also included a brief set of recommendations highlighting programmatic issues to address in the following quarter.

**FIGURE 2:**
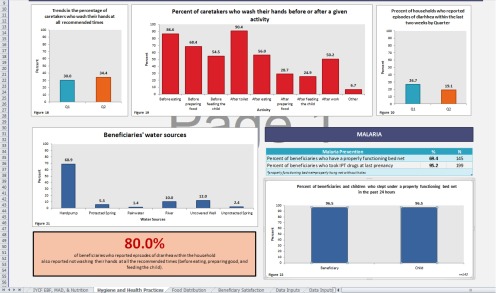
Example of LAUNCH's Excel-Based Dashboard to Present Monitoring Findings

In January 2014, LAUNCH shifted away from presenting data in a written format and began using an Excel-based dashboard (Figure 2). The motivation to present the data in a more visual format was to enhance the use of quarterly program data, making the information more accessible and comprehensible at a glance. The dashboard was organized by program topic (e.g., women's nutrition, breastfeeding practices, hygiene) and included tables and graphs of key programmatic indicators across time periods. Staff could copy the graphs directly from Excel for use in presentations and other documents. The dashboards continued for 3 quarters until the outbreak of Ebola disrupted program activities.

LAUNCH began using an Excel-based dashboard to present data in a more visual format to make it easier for program managers to access and understand.

## FINDINGS

Throughout the first 6 quarters of the nutrition and health monitoring system, more than 2,700 participants were interviewed at the FDPs (Table 1). Food monitors consistently reached the quarterly goal of approximately 400 interviews and maintained an even distribution of target groups, despite frequent staffing shortages and excluded FDPs due to canceled distributions as a result of inclement weather or poor road conditions. However, during the final 3 quarters, when data collection shifted to the household level, food monitors had substantial challenges reaching the desired targets. Reasons provided by food monitors for missing the targets consisted of logistical challenges in reaching the selected households, including time and transportation constraints and the absence of the selected household member. Quarter 9 coincided with the outbreak of Ebola, thereby affecting some program activities including data collection.

**TABLE 1.** Number of LAUNCH Program Participants Interviewed per Quarter by Point of Contact, Liberia, April 2012–June 2014

Quarter	Point of Contact			
	No. of Pregnant Women	No. of Lactating Women	No. of Mothers of Children <2	Total
**Food Distribution Points**				
Quarter 1 (Apr–Jun 2012), Lean Season	157	152	225	534
Quarter 2 (Jul–Sep 2012), Lean Season	79	105	221	405
Quarter 3 (Oct–Dec 2012), Non-Lean Season	69	116	210	395
Quarter 4 (Jan–Mar 2013), Non-Lean Season	109	112	189	410
Quarter 5 (Apr–Jun 2013), Lean Season	104	163	249	516
Quarter 6 (Jul–Sep 2013), Lean Season	81	144	282	507
**Households**				
Quarter 7 (Oct–Dec 2013), Non-Lean Season	6	58	116	180
Quarter 8 (Jan–Mar 2014), Non-Lean Season	13	74	122	209
Quarter 9 (Apr–Jun 2014), Lean Season	4	21	66	91

Note: Lean season refers to the period from April to September; non-lean season to the period from October to March.

LAUNCH made programmatic adjustments in response to findings from the monitoring system; these changes were then reflected in subsequent quarterly trends, indicating that the availability of timely data enabled the project to react quickly to issues and adapt activities appropriately. For example, in quarter 1 (April–June 2012), the findings revealed low participation in Care Groups, mother groups, and farmer groups. In the following quarters, there was a continual increase (more than doubling) in participation in these groups, from 30% to 80% and from 9% to 40%, respectively. This increase reflects an adjustment made by management, using real-time data, to actively focus on fostering better integration between food distribution and community programs (Table 2). Similarly, as the program scaled up food distribution and expanded the number of food recipients, the system provided evidence to increase the number of FDPs over time (from 9 to 14) and confirmed that their locations were well placed with relatively short distances for recipients to access, a project priority to make food distribution easily available.

Participation in Care Groups and mother and farmer groups more than doubled between the first and subsequent quarters, reflecting project adjustments made using real-time data from the mobile-based monitoring system.

**TABLE 2.** Attendance at LAUNCH Community Groups and Travel to Food Distribution Points, by Quarter, Liberia, April 2012–June 2014

	% Participating in Care Group or Mother Group^a^	% With a Family Member Participating in Farmer Group	% Traveling More Than 1 Hour to FDP
Quarter 1	30	9	30
Quarter 2	42	15	38
Quarter 3	46	18	24
Quarter 4	60	20	25
Quarter 5	91	31	28
Quarter 6	78	41	16
Quarter 7^b^	98	25	11
Quarter 8^b^	92	29	27
Quarter 9^b^	98	30	14

Abbreviations: FDP, food distribution point; LAUNCH, Liberia Agricultural Upgrading Nutrition and Child Health project.

^a^ Participation in one of these groups was a prerequisite for receiving food rations. A mother group is a group of women that meets under the leadership of one lead mother. A Care Group is a group of lead mothers (around 20) that participates in training.

^b^ In quarters 7, 8, and 9, the point of contact shifted to household level.

**FIGURE 3:**
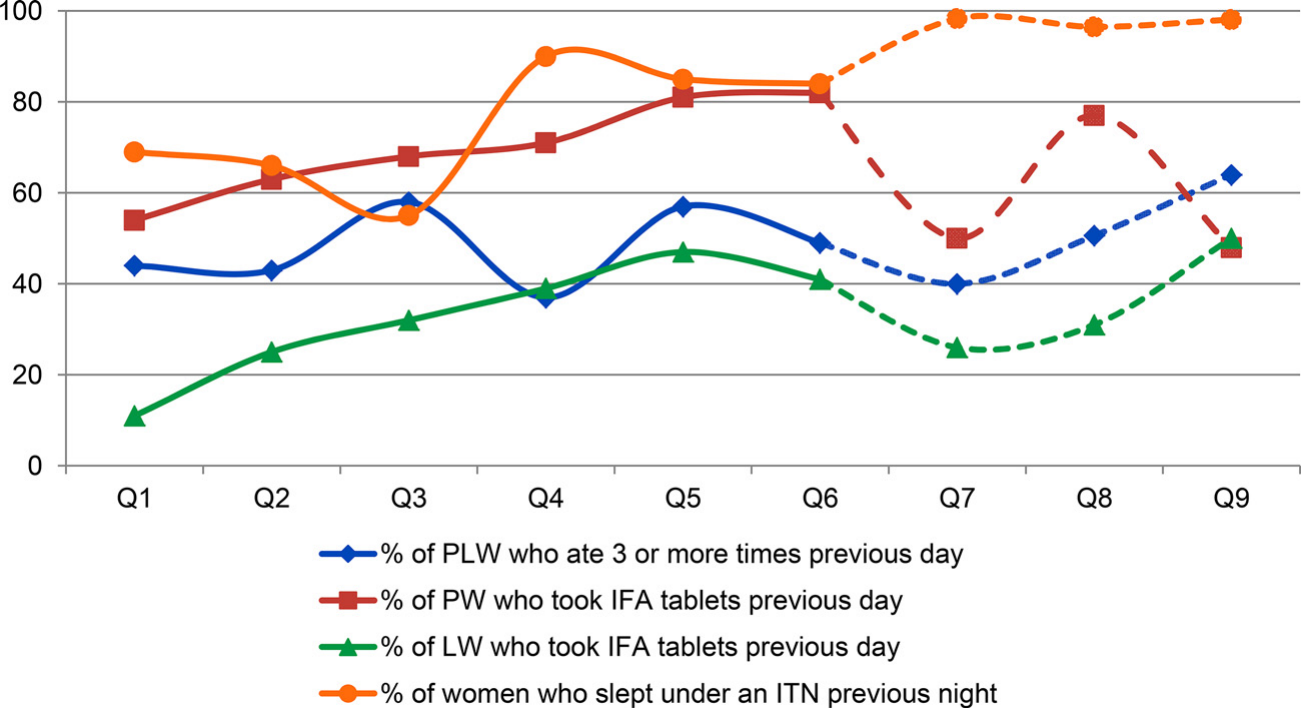
Findings from LAUNCH's Nutrition and Health Monitoring System: Women's Nutrition and Health, by Quarter, Liberia, April 2012–June 2014 Abbreviations: FDP, food distribution point; IFA, iron-folic acid; ITN, insecticide-treated net; LW, lactating women; PLW, pregnant and lactating women; PW, pregnant women.

Likewise, the system captured trends in key outcome indicators such as breastfeeding and complementary feeding practices, linking them to project activities as well as external factors such as seasonal changes and national health campaigns. The percentage of pregnant and lactating women who reported taking iron-folic acid supplements rose steadily over time, from 42% to 81% among pregnant women and from 10% to 40% among lactating women (Figure 3). This progress directly reflected project messages, through training of health care providers on Essential Nutrition Actions and directed Care Group activities about the importance of attending antenatal and postnatal visits and receiving iron-folic acid supplements. Likewise, the use of insecticide-treated mosquito nets by pregnant or lactating women decreased in the first 3 quarters of data collection, but subsequently increased over the following quarters. This increase happened concurrently with the relaunch of a nationwide campaign by the government to distribute insecticide-treated nets to pregnant women at their first antenatal care visit.

The percentage of pregnant and lactating women who reported taking iron-folic acid supplements rose steadily over time.

**FIGURE 4:**
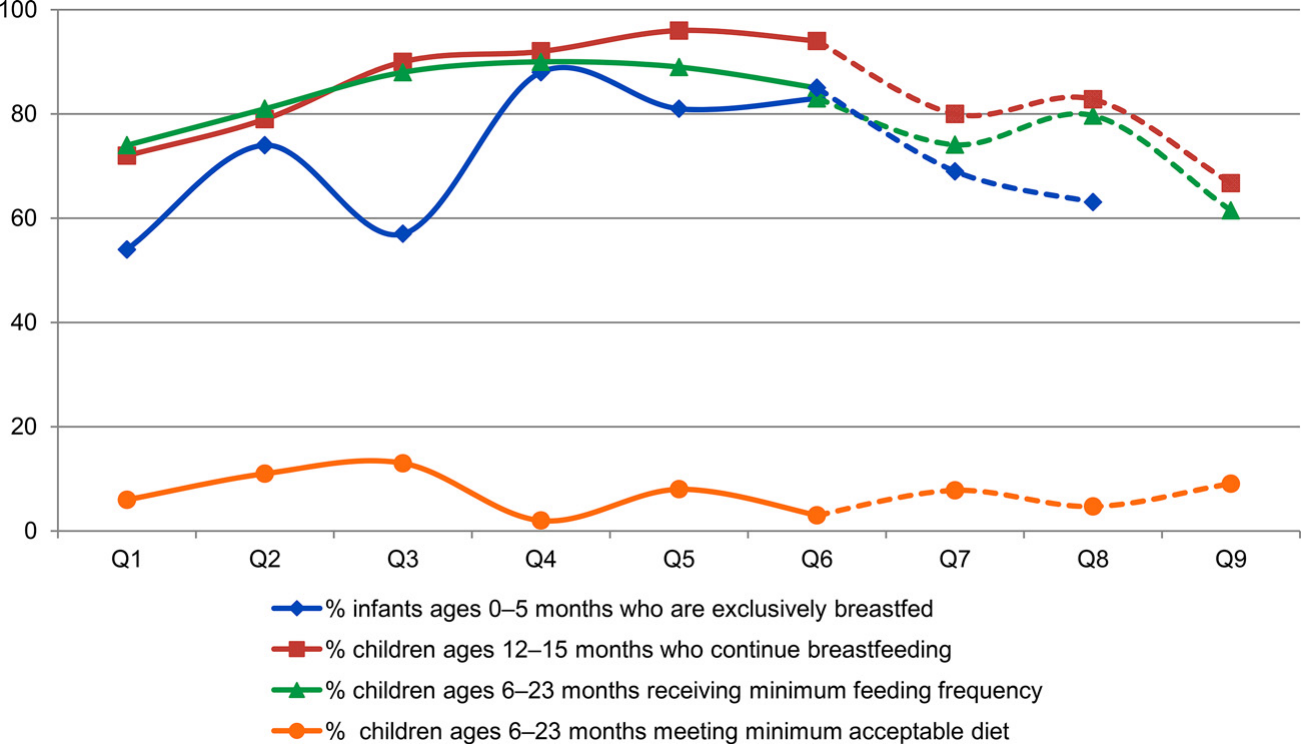
Findings from LAUNCH's Nutrition and Health Monitoring System: Infant and Young Child Feeding Practices, by Quarter, Liberia, April 2012–June 2014 Abbreviations: FDP, food distribution point; MAD, minimum acceptable diet.

A key component of the project was to promote and support complementary feeding practices with breastfeeding, with particular focus on fostering a diversified diet. The minimum acceptable diet estimated for breastfeeding and non-breastfeeding children remained low, with important variations across quarters (Figure 4). Minimum acceptable diet measures, for both breastfeeding and non-breastfeeding children ages 6–23 months, consider both feeding frequency and diversity; the barrier in achieving this indicator is mainly due to inadequate dietary diversity. Notably, similar to women's diet, complementary feeding practices peaked in the third quarter during the post-harvest period.

## DISCUSSION

In this era of using data-driven approaches, simple and cost-effective solutions to routine program monitoring are important to program success. Using real-time data designed to be collected in a routine and systematic manner is an innovative approach to applying information for better, more flexible program management and assessing nutritional trends and outcomes. The LAUNCH experience demonstrates that programmatic matters, such as enrollment in community groups and the number of needed delivery points, can be improved by regularly and routinely tracking program data. Furthermore, while LAUNCH ceased after its project cycle, we expect that other projects will build on this experience and include real-time data collection in their management system.

The routine data also provided timely information and insight about the progress of project outcomes (e.g., pregnant or lactating women receiving iron-folic acid supplementation, practicing exclusive breastfeeding, and continuing breastfeeding) and alerted managers when stagnation in food diversity and minimum acceptable diet occurred. The increased frequency of available data shed light on variations that occurred among program participants across quarters. Understanding these changes—often due to seasonal differences or external matters—over time enabled program management to quickly adapt and better address food insecurity, health, and nutrition components. For example, while trends in breastfeeding practices (i.e., exclusive breastfeeding and continued breastfeeding) showed an overall positive trend throughout the project cycle, variations between quarters were evident. These variations were likely due to the project cycle of increasing (or decreasing) intensity in promoting and supporting breastfeeding at the health facility and community levels. More specifically, the drop in exclusive breastfeeding in the third quarter could be explained by women being busier during harvest time in October and November, whereas the sustained increase in the following quarters is consistent with the percentage of women participating in the Care Groups.

The routine data provided timely information and insight about the progress of project and alerted managers when stagnation in food diversity and minimum acceptable diet occurred.

All too often systems fail for reasons that include funding scarcity, training shortages, lack of ownership, ineffective supervision, staff workload pressures, and a lengthy and confusing approach to entering data into the system.^11^ From the onset, the design of the nutrition and health monitoring system aimed to avoid many of these potential liabilities. By integrating routine data collection into project field staff responsibilities, thereby avoiding the need for additional staff and funding for implementation, the data could be collected in a more affordable and efficient manner with limited start-up costs. The tool itself collected the bare minimum information necessary for calculating key nutritional and programmatic indicators, thereby reducing burden and interviewer fatigue. Finally, the project provided continuous supervision and refresher trainings for staff using digital data collection as part of their dailytasks.

Integrating routine data collection into project field staff responsibilities enables the data to be collected in a more affordable and efficient manner with limited start-up costs.

The digital data collection platform, Magpi, was also instrumental in the process, as it improved routine programmatic functions by providing timely data for better tailored decision making as the project evolved. The use of mobile phones and a cloud-based server allowed for real-time quality data collection in otherwise hard-to-reach project locations by reducing the time and resource burden of paper forms and data entry. Data were automatically cleaner as the advanced programming forced all required questions to be answered in the correct format and skipped non-applicable questions. Using mobile phones also proved to be a great motivator for LAUNCH staff and program participants sitting for interviews who were eager to learn about the technology. Applying a digital data collection approach, however, does not remove certain manual tasks also associated with paper-based data collection activities—data processing and descriptive data analysis were still necessary to present the quarterly data. Additionally, issues related to working in areas with suboptimal connectivity and processes aimed at protecting the electronic equipment must be considered and integrated into the system design and training.

The use of mobile phones and a cloud-based server allowed for real-time quality data collection in otherwise hard-to-reach project locations by reducing the time and resource burden of paper forms and data entry.

Like all other routine monitoring systems, the validity and power of the results are only as good as the quality and consistency of the data. The routine component of the nutrition and health monitoring system consisted of conducting continuous quarterly surveys that were primarily successful when using the fixed delivery points to connect with program participants and conduct interviews. Staff easily reached their monthly interview targets even with other responsibilities on food distribution days. As a result, the indicators could be calculated accurately, using an appropriate number of program participants, and thus provide managers with statistically representative information for programmatic decision making on a quarterly basis. When the point of contact was shifted to the household level, however, staff fell short of their targets. Competing priorities for staff time, along with logistical issues such as transportation and reaching households in remote locations, resulted in far fewer interviews. This reduced the overall statistical power of results for both programmatic decision making and monitoring nutritional outcomes. Staff also struggled to locate pregnant women (6 in quarter 7, 13 in quarter 8, and 4 in quarter 9), undermining the ability of the system to monitor the nutritional practices of this vital group.

When the point of contact shifted from the individual to the household level, staff fell short of their interview targets.

In addition, the FDPs were a reliable location for interacting with program participants. In a February 2013 post-distribution monitoring assessment, the vast majority of program participants (85% of respondents) reported collecting the food themselves rather than sending an alternate. The concerns, therefore, of the monitoring system missing out on significant portions of program participants by automatically excluding alternates did not represent a strong argument against the FDP as an appropriate point for data collection. While household-level surveys are still important for project evaluation purposes, as well as for tracking direct observational based indicators, the cost savings of using fixed points of contact, including staff time and fuel for transport, as well as the logistical ease, should outweigh concerns of selection bias when collecting good-quality monitoring data.

Finally, displaying data in an easily digestible format was important for the utility of the system. The data dashboards quickly and easily conveyed program achievements and helped managers discern important programmatic information for decision making at a glance. Dashboards also assisted program staff to engage with data visually, allowing for more interaction and greater insights into program results. Displaying key program indicators visually improved data use and empowered LAUNCH staff to use their quarterly data for decision making.

Visually displaying key program indicators improved data use for decision making.

## CONCLUSIONS

Digital data collection platforms can play a vital role in improving routine programmatic functions. LAUNCH's experience using mobile technology to improve project management and to assess women's and children's nutrition-related progress has been successful and presents a model for managing and improving program implementation of similar projects. Fixed gathering points, in our example FDPs, represent an opportunity to easily access participants and allow managers to identify strengths and weaknesses in project implementation. For programs that track individuals over time, a mobile tool combined with a strong database can greatly improve efficiency and data visibility and reduce resource leakages.

## Notes

**Peer Reviewed**
